# Virtual Reality–Based Rehabilitation as a Feasible and Engaging Tool for the Management of Chronic Poststroke Upper-Extremity Function Recovery: Randomized Controlled Trial

**DOI:** 10.2196/37506

**Published:** 2022-09-27

**Authors:** Alejandro Hernandez, Liudmila Bubyr, Philippe S Archambault, Johanne Higgins, Mindy F Levin, Dahlia Kairy

**Affiliations:** 1 Centre for Interdisciplinary Research in Rehabilitation Montreal, QC Canada; 2 Independent biostatistician Montreal, QC Canada; 3 School of Physical & Occupational Therapy McGill University Montreal, QC Canada; 4 Ecole de sciences de la réadaptation Université de Montréal Montreal, QC Canada

**Keywords:** rehabilitation, serious game, stroke, telerehabilitation, upper extremity, virtual reality–based rehabilitation, virtual reality, virtual care

## Abstract

**Background:**

A growing number of stroke survivors are left with little to no rehabilitation services upon discharge from stroke rehabilitation, although arm deficits may persist or develop from disuse once rehabilitation services have ceased. Virtual reality (VR)–based rehabilitation, combined with new technologies such as telerehabilitation, including serious games using VR environments that encourage users to practice functional movements from home with minimal supervision, may have an important role to play in optimizing and maintaining upper extremity (UE) function.

**Objective:**

The primary objective of this study is to determine the extent to which a 1-month intervention using a VR-based serious game is effective in improving UE function compared with an evidence-based home exercise program. A secondary objective is to assess the feasibility of implementing the intervention for chronic stroke rehabilitation in participants’ homes.

**Methods:**

A total of 51 chronic stroke participants were randomized to treatment (n=26, 51%; Jintronix system) or standard care (n=25, 49%; standardized Graded Repetitive Arm Supplementary Program kit home program) groups. The participants were evaluated at baseline (before), immediately after the intervention (after), and at follow-up (4 weeks). The primary outcome measure was the Fugl-Meyer Assessment for UE (FMA-UE). Secondary outcome measures included the Stroke Impact Scale and an abridged version of the Motor Activity Log-14. Self-reported number of sessions was logged for the standard care group.

**Results:**

No statistically significant differences between groups were found across measures. Overall time effects were found for the FMA-UE (*P*=.045), specifically between preintervention and postintervention time points for both groups (*P*=.03). A total of 9 participants in the treatment group reached or surpassed the minimal clinically important difference in scores for the FMA-UE, with 7 (78%) of them having baseline low or moderate arm function, compared with 3 (33%) participants in the standard care group. Furthermore, 56% (9/16) of the participants in the treatment group who actively engaged with the system reached the minimal clinically important difference for the FMA-UE, compared with none for the 0% (0/10) less-active participants.

**Conclusions:**

These findings suggest that UE training for chronic stroke survivors using virtual rehabilitation in their home may be as effective as a gold standard home exercise program and that those who used the system the most achieved the greatest improvement in UE function, indicating its relevance to being included as part of ongoing rehabilitation services.

**Trial Registration:**

ClinicalTrials.gov NCT02491203; https://clinicaltrials.gov/ct2/show/NCT02491203

**International Registered Report Identifier (IRRID):**

RR2-10.1016/j.cct.2015.12.006

## Introduction

### Background

As of 2019, there were over 400,000 stroke survivors in Canada alone, a number that is projected to double by 2040 [1]. Hemiplegia, or weakness of one side of the body, can often translate into loss of upper extremity (UE) function. Unfortunately, the rate of full recovery of the affected arm was found to be only approximately 40%, especially in more severe cases [2]. Furthermore, recent trends in health care delivery often result in a shorter length of stay for an increasing number of stroke survivors, in spite of persistent functional deficits [3]. Outpatient or home care services may provide some rehabilitation care for a short time after stroke, but they are limited by long distances to and from home, high travel costs, and limited availability of caregivers [4].

The Canadian Stroke Best Practices Recommendations, updated in 2019, provide guidance for the provision of rehabilitation services [1]. On the basis of the evaluation of a stroke survivor’s arm function, early treatment and individualized therapies of appropriate intensity and duration are recommended to optimize recovery in an inpatient clinical setting or on an outpatient basis, including during the chronic phase of stroke recovery. Providing intensive, meaningful, task-specific exercises to restore sensorimotor function is an important component of the recommended rehabilitation interventions, including traditional as well as more recent approaches, such as constraint-induced movement therapy and virtual reality (VR). VR provides an opportunity for the person to engage in repetitive movements and has been recognized as a valid complement to standard therapy [1].

The potential benefits of applying VR technology in physical rehabilitation notwithstanding, it is still unclear what therapy dosage levels are required to achieve optimal recovery, especially when considering factors such as time since stroke and severity of motor deficits. Dosage can be measured according to 3 distinct parameters: frequency, or number of exercise sessions per week; duration, or the period over which therapy is delivered; and number of repetitions, or time spent in active therapy, with an emphasis on the practice of challenging rather than overlearned tasks [5]. A study using motor learning methods as experimental interventions, observed statistically significant improvements in arm function after 300 hours of arm therapy practice over a 12-week period [6]. Furthermore, it would seem that the right combination of sufficiently high dosage and intensity training may be key to maintaining UE gains over the long term [7].

The provision of remote rehabilitation solutions has varied greatly over the years in their levels of technological sophistication, whether through simple telephone communications or more complex videoconferencing solutions and finally toward the more recent development of sensor and remote monitoring technologies that enable web-based applications to be deployed in the home [8]. Telehealth is an emerging technology that enables remote communication between patients and health professionals across health care fields, such as physical rehabilitation [9]. Communication can occur in real time through secure web-based platforms, allowing for face-to-face meetings. It may also occur asynchronously with therapists and patients logging onto platforms at different times to exchange relevant information. Telerehabilitation—or telehealth in the context of rehabilitation—could increase access to rehabilitation services by allowing for the remote supervision of patients who would otherwise be ineligible for or unable to access rehabilitation services following discharge. A systematic review of studies on motor recovery after a stroke suggests that interventions via telerehabilitation can be as effective as conventional in-person therapy [10].

As a complement to telerehabilitation, novel clinically oriented video gaming consoles, often referred to as serious games, are becoming increasingly accessible in health care settings. VR-based rehabilitation is increasingly accepted in the clinical setting for engaging patients to perform exercises and tasks repeated many times, which is the main principle of practice in standard care after stroke UE rehabilitation [11]. Older, widely available commercial platforms such as the Nintendo Wii gaming console were designed to physically engage the user in sport-like activities. A literature review examining the feasibility and effectiveness of commercial gaming consoles found that all 10 studies using Nintendo Wii as an intervention program showed gains in functional UE measures [12]. This promising finding suggests that Nintendo Wii and other similar VR-based serious games could be used to support recovery efforts in the clinical setting, although it remains to be verified whether their role can go beyond serving as adjuncts to standard therapy [13]. Not all VR serious game systems are equipped with a telehealth feature enabling participants to communicate remotely with a therapist; those that do provide a unique opportunity to customize rehabilitation interventions.

The Jintronix (Jintronix Inc) gaming console was designed to engage stroke survivors to recover lost UE function through a series of interactive games that encourage repetitive arm movements. A pilot study using the Jintronix system in a 2-arm randomized clinical trial with an acute poststroke clientele concluded that the VR gaming console was safe and feasible in its capacity to complement traditional therapy [14]. A meta-analysis concluded that similar home-based telerehabilitation approaches were as feasible as usual care [15]. Other studies using home-based interventions have reported modest UE gains in chronic poststroke clientele [16,17].

### Objectives

First conceived as a support tool for stroke survivors, the Jintronix system presents itself as a promising tool to allow poststroke patients to pursue their UE rehabilitation, but who are no longer receiving standard care in the months or years since their discharge. At the onset of our study, no previous studies have investigated the use of the Jintronix system as a remotely supervised home-based program for UE rehabilitation in chronic poststroke clientele. Therefore, the primary aim of this study was to assess the efficacy of a month-long home-based Jintronix system intervention in promoting UE functional recovery in chronic poststroke patients no longer receiving rehabilitation services. In line with the home-based nature of the intervention, as a secondary aim, we examined the feasibility of implementing the system in the homes of chronic stroke survivors.

## Methods

### Study Design

A single-blind (evaluator-blinded) parallel, 2-arm randomized controlled trial with a before, after, and follow-up design was used for this study in a chronic stroke population [18].

### Ethical Considerations

This study was granted ethics board approval by the Research Ethics Board of the Centre for Interdisciplinary Research in Rehabilitation of Greater Montreal (CRIR-937-0214). All participants provided informed consent before participating in the study.

### Recruitment

A block randomization strategy with a block size of 6 using a random number generator was carried out by the study coordinator to randomly allocate participants into 1 of 2 distinct intervention groups. Sealed envelopes containing the group’s identity were sequentially numbered according to initial randomization by block order. Allocation was performed previously but only revealed following the first in-person evaluation by the study coordinator (participants could not be blinded to the group assignation). Each intervention consisted of a 4-week long program, which was broken down as follows:

1. Treatment: home-based exercise program via the Jintronix system monitored offline by a therapist.
2. Standard care: home-based exercise program manual (Graded Repetitive Arm Supplementary Program [GRASP]) provided by a therapist without further supervision.

Textbox 1 outlines the participation inclusion and exclusion criteria.

**Participant inclusion and exclusion criteria.**

**Inclusion criteria**

- First-time stroke having occurred >6 months earlier
- Having residual mild to moderate upper extremity (UE) impairments with a 2 to 6 score on the Chedoke-McMaster arm component (a quick screening tool used to ensure an adequate level of movement for the program) [19]
- No longer receiving rehabilitation services

**Exclusion criteria**

- Insufficient motor control to move the avatar onscreen
- Visual or auditory deficit
- Inability to understand simple verbal instructions
- Insufficient sitting balance
- Shoulder pain or pre-existing UE impairment limiting arm movement

### Home-Based Intervention

**Figure 1:**
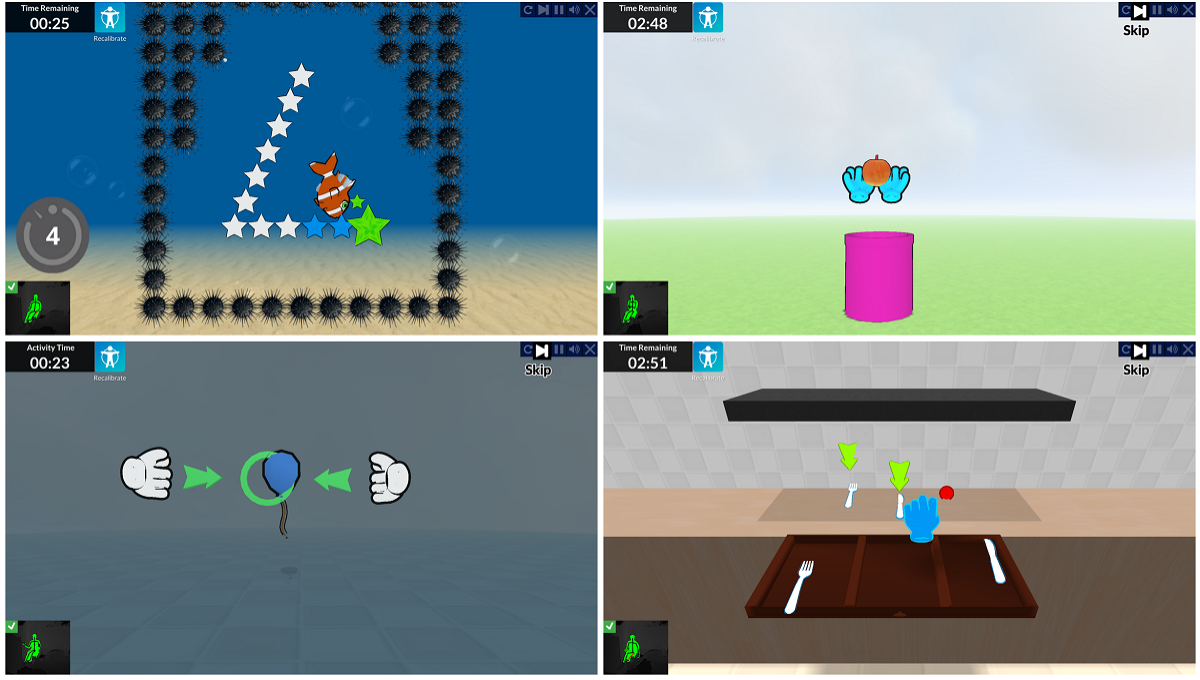
The Jintronix system. Clockwise from top left: Fish Frenzy, Catch-Carry-Drop, Kitchen, Pop Clap game activities.

The Jintronix system—composed of the Jintronix software installed on a computer, a large screen, and a Microsoft Kinect depth-detecting infrared sensor camera—was connected to the web via the participant’s internet service provider, or through an internet key we provided if no service was installed. The Kinect camera tracks limb movements within a 3-meter range in 3D space without the need for a handheld controller. Data extracted by the camera are transferred in real time to the Jintronix software, which outputs a display of an avatar onscreen reflecting the user’s movements. For example, the kitchen activity invites the user to reach a target placed in a virtual 3D kitchen setting; another activity requires bilateral movements of the arms to catch, carry, and drop objects in a 2D plane (Figure 1). The purpose of the system was to engage participants in repeated unilateral and bilateral UE movements to achieve satisfactory game scores needed to progress through the difficulty levels. User performance statistics such as movement speed and accuracy as well as overall game score could be accessed from the company servers.

Participants randomized into the treatment group first set up a short meeting with a trained physiotherapist who provided them with a brief tutorial on the use of the Jintronix system and later set up a time with a technician to schedule the system’s home installation. Participants were recommended to follow the program 5 times a week for ≥20 minutes per session. Taking into account the baseline level of UE function, the therapist custom tailored a simple program for each participant. Program progress was remotely monitored through the Jintronix system once to twice a week, and the level of difficulty, speed, and trajectories of arm movements were remotely and asynchronously adjusted by the therapist to maintain an optimal challenge according to the participant’s UE improvement throughout the intervention.

The standard care group participants were provided with a manual for a standardized exercise program, the GRASP. The GRASP has been found to be effective as a supplement to ongoing UE rehabilitation during subacute stroke [20] or as a treatment alternative for discharged chronic stroke patients [21]. No therapists supervised participants’ progress in the standard care group. A meeting was arranged with each participant before beginning of intervention to cover the components of the program. Participants were encouraged to engage in the program as many times a week as possible. Participants self-reported the number of sessions completed at the end of the 4 weeks. Although the GRASP program may have shared some similarities with the treatment (Jintronix system) program, such as promoting a variety of movements of the elbow joint, it also promoted wrist joint and hand dexterity exercises that were not included in the treatment program. Above all, a significant difference between the 2 groups lay in the provision of the program content: the treatment group program provided a very interactive visual and auditory experience, whereas the standard care group program provided the user with a simple manual in booklet form, as is often provided in outpatient care.

### Outcome Measures

Baseline demographics were collected for all participants on their first visit. A total of 3 clinical efficacy outcome measures were selected to assess functional changes in the upper limb. The primary clinical outcome measure consisted of the Fugl-Meyer Assessment for UE (FMA-UE), which quantifies UE impairment after stroke. A gold standard in clinical practice, it has high interrater reliability and content validity [22] and is widely used across a range of clinical studies targeting poststroke recovery [23]. The Stroke Impact Scale (SIS; SIS 3.0; along with its individual Strength, activities of daily living [ADL], Mobility, and Hand Function components) and the Motor Activity Log (MAL; MAL-14, abridged 14-question version) were used as secondary outcome measures to self-assess quality of life and the use of the impaired arm in ADL, respectively. Both outcomes were chosen for their strong internal consistency and test-retest reliability [24,25].

To assess the feasibility of implementing the Jintronix system at home, several variables were collected, among which are the following: time of home installation, number of sessions throughout the 4-week intervention period, total time spent on the program, pain and fatigue indicators, and episodes of dizziness or falls.

Evaluations using these clinical outcome measures were carried out at baseline (before), after the intervention (after), and at 4-week follow-up (follow-up). Evaluators were blinded to participant group allocation and were not involved in the interventions.

### Statistical Analysis

#### Overview

Demographic variables exhibiting normal data distribution were represented by their means and SDs. Median and IQRs were used when describing data that were not normally distributed. Unlike the mean, the median is more robust against the effect of potential outliers in overall as well as subgroup analyses [26]. Effects were tested against a significance level of Cronbach α=.05. When available, the minimal clinically important difference (MCID) was used as a cutoff to determine clinically meaningful differences.

Normality of outcome distributions was assessed using the Kolmogorov-Smirnov test (*P*>.05 for normality) to investigate further differences in the groups. Either 2-sample *t* tests or Wilcoxon 2-sample tests were conducted depending on the normal or nonparametric nature of the distributions, respectively.

Clinical data collected from onsite assessments as well as data recorded from the Jintronix system were stored in a secure database REDCap (Research Electronic Data Capture; Vanderbilt University). Statistical analyses were carried out using the SAS 9.4 (SAS Institute) software package.

#### Sample Size

A sample size of 26 participants per group was determined using G*Power, assuming a medium effect size of 0.2, accounting for a 20% attrition rate, and setting the Cronbach α to .05 and the power to 0.8.

#### Modeling

A mixed model paradigm with a compound symmetry structure was used to model the analyses. The group and time variables were set as factors to account for between-participant and within-participant differences. An adjustment for baseline differences in variables such as participant age and arm function at the onset of participation was performed to correct for improprieties in baseline characteristics from the observed data, thus making the compared groups more homogeneous.

#### Subgrouping Analyses

The *baseline FMA-UE score* was chosen as a subgroup factor to further explore its role in participant improvement across outcome measures. The FMA-UE cutoff scores were used to define the factor’s 3 levels: low, moderate, and high function. The choice of cutoff scores was based on previous studies examining the FMA-UE as a factor [27,28]. Similarly, to further explore efficacy, *active playing time* was used to create a 2-level factor to categorize treatment group participants around the 400-minute cutoff time, as recommendations were for participants to engage in five 20-minute sessions per week (totaling 400 minutes for the entirety of the 4-week program).

## Results

### Participant Demographics

**Figure 2:**
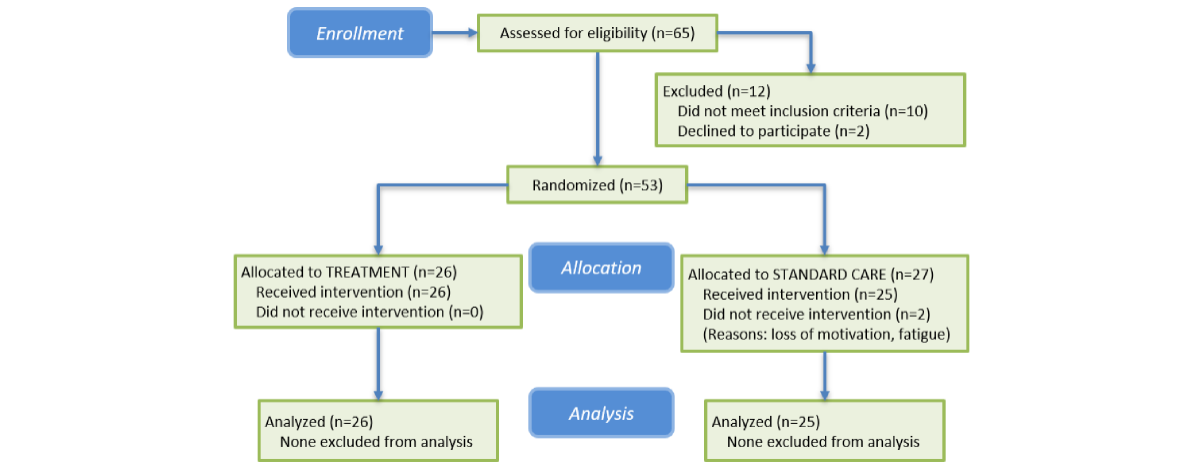
Flow diagram of the study’s enrollment process.

A total of 53 chronic poststroke individuals consented to participate in this study. Of the 53 participants, 2 (4%) withdrew following consent and randomization into the standard care group, with reasons cited being loss of motivation or fatigue; 51 participants completed the study (n=26, 51% and n=25, 49% for the treatment and standard care groups, respectively). A participant enrollment flow diagram is presented in Figure 2.

In total, 27% (14/51) of the participants were female, with group ratios differing slightly (9/26, 35% for treatment and 5/25, 20% for standard care). Mean participant age was 59.8 (SD 13.1) years for treatment and 56.7 (SD 11.2) years for standard care. Median time since stroke was 63 months (IQR 5.3 years) and 53 months (IQR 4.4 years) for the treatment and standard care groups, respectively. There were no statistically significant differences between groups for the list of relevant participant demographics, as outlined in Table 1.

**Table 1:** Participant demographics at baseline (before the intervention).

Variables			Treatment (n=26)		Standard care (n=25)
Age (years), mean (SD)			59.8 (13.1)		56.7 (11.2)
Male, n (%)			17 (65)		20 (80)
**Stroke type, n (%)**					
	Ischemic	14 (54)		10 (40)	
	Hemorrhagic	7 (27)		7 (28)	
	Unknown	5 (19)		8 (32)	
**Handedness, n (%)**					
	Left	2 (8)		3 (12)	
	Right	23 (88)		22 (88)	
	Ambidextrous	1 (4)		0 (0)	
Left-side hemiparesis, n (%)			13 (50)		11 (44)
Dominant side affected, n (%)			15 (58)		13 (52)
**Time since stroke, median (IQR)**					
	In years	5.3 (1.5-8.1)		4.4 (2.2-7.4)	
	In months	63 (18-97)		53 (26-89)	
Montreal Cognitive Assessment score, median (IQR)			25 (20-27)		25 (24-27)
Chedoke-McMaster score, median (IQR)			4 (3-5)		4 (3-5)
Fugl-Meyer Assessment for upper-extremity score, median (IQR)			30 (17-52)		38 (22-55)

### Overall Group Analyses

Mixed model analysis (adjusted for baseline differences) revealed no overall statistically significant differences between groups across all outcome measures (Table 2). However, there was a significant time effect for the FMA-UE (*P*=.046) and SIS-total (*P*=.048) outcome measures. In particular, for the FMA-UE, a significant time effect was observed in the before to after periods (*P*=.03) but not for the other periods, including between after and follow-up (and before and follow-up). Although the FMA-UE trended toward better scores between before and after periods (*P*=.08), no group-by-time interactions were found to be statistically significant across any of the measures.

**Table 2:** Mixed models results across outcome measures by effect type.

Outcome measure		Group		Time		Group×time	
		*F*test (*df*)	*P* value	*F*test (*df*)	*P* value	*F*test (*df*)	*P* value
Fugl-Meyer Assessment for upper extremity		1.50 (34)	.23	3.19 (86)	.046	2.62 (86)	.08
**Motor Activity Log**							
	Amount	0 (34)	.98	.30 (86)	.74	.17 (86)	.85
	Quality	0 (34)	.99	1.00 (86)	.37	.03 (86)	.97
**Stroke Impact Scale**							
	Strength	1.81 (32)	.19	1.89 (80)	.16	0.04 (80)	.96
	Activities of daily living	0.84 (32)	.37	0.94 (78)	.40	2.42 (78)	.09
	Mobility	0.80 (34)	.38	1.67 (85)	.19	1.52 (85)	.22
	Hand function	0.48 (34)	.49	0.94 (86)	.39	0.80 (86)	.45
	Total	0.09 (27)	.76	3.17 (72)	.048	2.14 (72)	.12

Outcome measure distributions were mostly observed to exhibit nonnormal distributions, with the exceptions of SIS-ADL and SIS-mobility, which were normally distributed (*P*=.15 and *P*=.12, respectively). The 2-sample Wilcoxon tests revealed no statistically significant differences between groups across time points (before, after, and follow-up). No significant differences were observed in the results of the 2-sample *t* tests carried out on the SIS-ADL and SIS-mobility measures.

**Figure 3:**
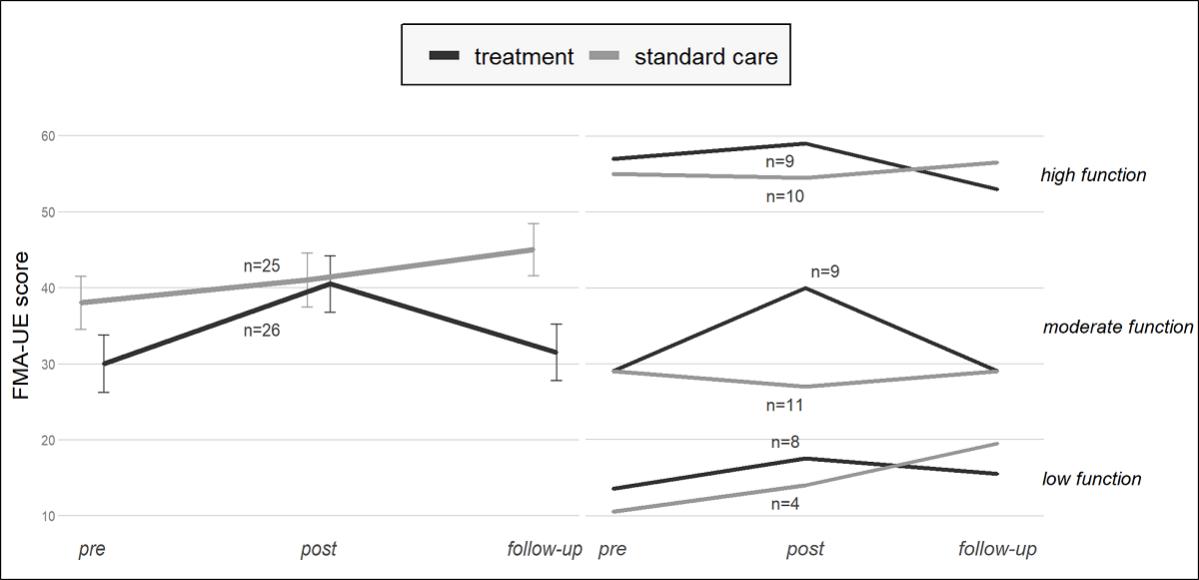
Left: median Fugl-Meyer Assessment for upper-extremity (FMA-UE) score over time by group; right: by group and baseline arm function.

The groups’ median FMA-UE scores over time are shown in Figure 3 (left-hand side). The upward trend between the before and after time points tends to corroborate the significant time effects identified. Gains obtained by the treatment group after the intervention were no longer seen at follow-up, as seen in Figure 3 (right-hand side).

### Subgroup Analysis by Baseline FMA-UE

Mixed model analyses using baseline FMA-UE as a subgroup factor revealed a statistically significant difference between groups for the SIS-strength measure, but only for the high-function subcohort (*P*=.046). Post hoc analyses of SIS-strength scores after the intervention revealed a significant difference between the groups (*P*=.008). However, no statistical differences were found at the follow-up. No other SIS component measure (ADL, mobility, or hand function) produced significant differences between groups or within the subgroup analyses.

The groups’ median FMA-UE scores per baseline FMA-UE are shown in Figure 3 (right side). Although the differences were not statistically significant, an 11-point change in the median FMA-UE score was observed before and after the intervention for the treatment with the moderate FMA-UE subgroup (n=9 participants). This was the only subgroup that surpassed the 5-point MCID for the FMA-UE measure.

The number and proportion of participants across both groups that either reached or surpassed the MCID threshold (positive 5-point FMA-UE change in score) are displayed in Table 3. Nearly half of the participants (7/17, 41%) in the low- and moderate-function subcohort from the treatment group reached the MCID, whereas approximately a fifth of the participants (3/15, 20%) were observed for the same subcohort from the standard care group. Overall, 35% (9/26) of the treatment group participants achieved scores at or above the MCID, a little under double the ratio seen in standard care participants (5/25, 20%), although a chi-square analysis did not support these rates as statistically significant (*P*=.32).

**Table 3:** Number of participants having reached or surpassed the minimal clinically important difference (MCID) on the Fugl-Meyer Assessment for upper extremity (FMA-UE) after intervention, according to group and baseline arm function. Number and proportion of treatment group participants having reached or surpassed the MCID on the FMA-UE after the intervention, by levels of gameplay time and baseline arm function.

Group	Baseline FMA-UE arm function level, MCID/n^a^, %^b^				Totals, MCID/n, %
	Low	Moderate	High		
Treatment	3/8, 38	4/9, 44	2/9, 22	9/26, 35	
Standard care	1/4, 25	2/11, 18	2/10, 20	5/25, 20	
<400 minutes	0/4, 0	0/2, 0	0/4, 0	0/10, 0	
>400 minutes	3/4, 75	4/7, 57	2/5, 40	9/16, 56	

^a^MCID/n: ratio of participants reaching MCID on total subgroup number.

^b^Ratio percentage.

### Intervention Group Feasibility and Efficacy

The key descriptive feasibility and efficacy findings for the treatment group participants are shown in Table 4. The standard care group participants engaged in the GRASP program for a median of 12 (self-reported) sessions over 4 weeks, with 50% (11/22) of participants ranging between 8 and 16 sessions. Participants in the treatment group engaged with the Jintronix system for a median of 21.5 sessions and invested a total duration of 527 minutes (with 13/26, 50% of participants ranging between 310 and 673 minutes). Of particular note were participants in the treatment group with moderate arm function: they tended to spend more time exercising (median 652 minutes) compared with the low and high functional participants. In addition, the more active participants gained a median of 5.5 points in their FMA-UE scores compared with 0 for the less-active participants and 1 for the standard care group.

**Table 4:** Treatment group participant statistics following a 4-week intervention.

		Population size, N	Number of sessions, median (IQR)^a^	Time (minutes), median (IQR)	Change in Fugl-Meyer Assessment for upper extremity, median (IQR)
Standard care		22^b^	12 (8 to 16)	N/A^c^	1 (−2 to 4)
Treatment		26	21.5 (16 to 27)	527 (310 to 673)	2 (0 to 6.8)
**By Fugl-Meyer Assessment for upper-extremity level**					
	Low	8	19 (16 to 22)	431 (237 to 660)	3 (0.5 to 6.8)
	Moderate	9	26 (22 to 30)	652 (479 to 864)	2 (0 to 9)
	High	9	21 (13 to 27)	468 (287 to 570)	0 (−1 to 3)
**By total duration**					
	<400 minutes	10	15 (11.5 to 16.8)	269 (152 to 317)	0 (−1 to 2.8)
	>400 minutes	16	26.5 (22 to 30.5)	648 (561 to 855)	5.5 (0 to 9)

^a^IQR expressed as (25th percentile-75th percentile).

^b^Data available for 22 of the 25 standard care group participants.

^c^N/A: not applicable.

The installation time on the Jintronix system at home ranged between 15 and 40 minutes. Participants in the treatment group reported median fatigue and pain scores of 3.3 and 1.8, respectively, both rated on a 10-point scale (with 10 representing the maximum). No adverse events, such as falls or episodes of dizziness, were reported by any participant. Two participants reported difficulties with the technology, primarily related to controlling the mouse and navigating the gaming interface.

**Figure 4:**
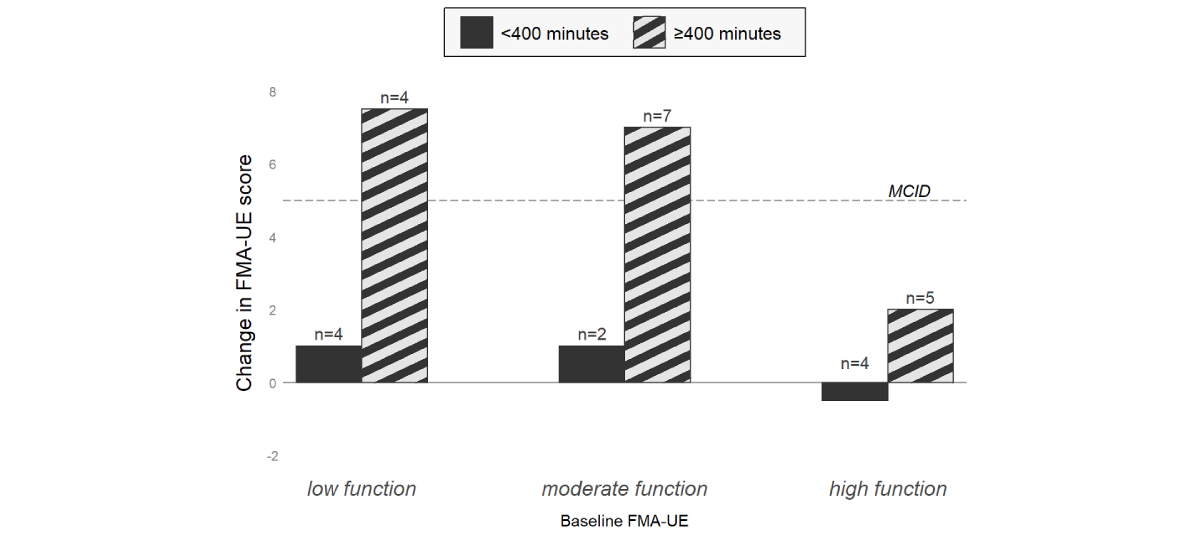
Change in median Fugl-Meyer Assessment for upper-extremity (FMA-UE) score after intervention for treatment group participants; by baseline arm function and level of gameplay duration. MCID: minimal clinically important difference.

The median change in FMA-UE score after the intervention is shown along a continuum of baseline arm function levels (Figure 4). A downward trend can be observed in the FMA-UE gains as the baseline arm function increased for the more active subgroup (diagonal patterned bars). Of note are the participants whose arm function were either low or moderate; both subgroups achieved a median of 7 or higher increase in the FMA-UE (above the 5-point MCID). By contrast, the dark bars suggest that participants having invested less than the recommended dosage tended to produce little or no gains regardless of baseline arm function. Despite the visual trends displayed in Figure 4, the differences were not statistically significant (*P*=.08 for the low function subgroup and *P*=.55 and *P*=.27 for the moderate and high subgroups, respectively).

Interestingly, none of the 10 less-active participants achieved the 5-point FMA-UE, which indicates a clinically important change, whereas 56% (9/16) of more active participants achieved significant gains in arm function (Table 3).

## Discussion

### Principal Findings

Within the context of this randomized controlled trial, we examined the efficacy and feasibility of a noninvasive VR-based rehabilitation serious game for UE training with a chronic poststroke clientele no longer receiving rehabilitation services. Participants in both the treatment and standard care groups were able to successfully engage in their assigned interventions. After accounting for baseline differences across participant characteristics, no statistically significant differences were found between groups across all outcome measures. However, both groups showed statistically significant improvements in the FMA-UE and SIS outcome measures over time, particularly between the periods before and after the interventions. Therefore, the program based on the Jintronix system was noninferior compared with the standardized GRASP program in improving UE function. All participants remained within the group to which they were assigned, thereby respecting the intention-to-treat principle, although not all participants achieved the recommended dosage. Therefore, further subgroup analyses were conducted to better understand the dosage administered to the treatment group.

### Amount of Time Played Makes a Difference

The more active treatment group participants improved on their FMA-UE scores by a median of 5.5 points, whereas their less-active counterparts gained a 0-point median change in score. Treatment group participants lost most gains acquired on the month following the end of intervention. Similar observations were noted in studies conducting UE treatment programs with chronic poststroke patients [29,30].

Findings from within the treatment group suggest that participants having invested more time engaging in the activity (Figure 4) seem to confirm that “the more one puts into one’s recovery, the more one gets out of it” [31]. This appears to hold true for both low and moderate FMA-UE subcohorts but less so for the high FMA-UE subcohort.

In addition to the amount of time spent exercising, there were important differences between programs that included more games conducive to repetitive movements of the shoulder and elbow joints by the Jintronix system, whereas the GRASP program included a considerable focus on wrist and finger movements. Therefore, the amount of time cannot be isolated from the rest of the intervention itself. However, the results of this study support those of previous studies which found that game-based rehabilitation systems could spark a greater interest in the participant, which could make it easier to spend more time on a program than usual care [32].

### Baseline Function Plays a Role in the Rate of Recovery

Subgrouping participants by level of arm function showed a trend toward differences in group scores in the moderate FMA-UE cohort (Figure 3), with an 11-point median FMA-UE score difference between groups (statistically nonsignificant). The data observed suggest minimal clinically meaningful changes in the treatment group. However, higher-functioning poststroke participants may have greater difficulty in obtaining greater gains, in part perhaps due to the ceiling effect of the outcome measure. A better understanding of the relationship between high function and the extent of improvement would be worthy of further exploration to best determine dosage. In spite of the clinical measure’s demonstrated content validity and reliability, the baseline FMA-UE score may be less responsive to change when it is already high to begin with [33]. Participants with low baseline FMA-UE had smaller gains than moderately functional stroke survivors but greater gains than the higher-functioning participants.

Kinematic measures could fill in the gaps where established clinical measures fail to detect changes. Rather than only quantifying functional improvement using a MCID threshold, a more nuanced approach could be envisioned for participants at the higher and lower ends of the UE functional spectrum. This could be implemented via wearable sensors or robot-mediated consoles, which measure variables such as the speed of movement, range of motion, and path smoothness [34]. Some studies have found significant correlations between kinematic measures and the FMA-UE, although they caution against substituting out established clinical measures [35]. A meta-analysis concluded that kinematic measures can be good complements to clinical outcome measures as they are apt for detecting smaller improvements [36]. Although kinematic variables were not collected (given that they were outside the scope of this study’s primary objectives), they may provide added value to future studies aimed at implementing similar technologies as an adjunct to clinical outcome evaluations.

Sensor technology may become more omnipresent in the future, tracking arm activity to accurately account for activity metrics performed within and beyond a prescribed intervention program [37]. It could also serve in the collection of kinematic measures during participant evaluation, especially if done remotely or when established clinical outcome measures fail to detect smaller changes in arm function. In fact, its hands-free simplicity of use prompted a study to verify and confirm its validity as a means of assessing UE function in a clinical setting [38].

### On the Question of Dosage

We provided simple participation guidelines formulated in such ways as “5 times per week, 20 minutes per session for 4 weeks,” in an effort to promote program engagement. This was based on prior examples of telerehabilitation intervention programs for upper-limb recovery after stroke [39,40]. A meta-analysis found that exercise dosage strongly predicted functional motor recovery when it was modeled as a linear regression of key predictor variables, such as dosage time and time since stroke [41]. This finding was also confirmed by a study that observed a linear relationship between dosage and functional outcome gains, but only up to a certain number of hours, beyond which the returns for any additional time tapered off [5].

Although on the one hand, our results showed nonretention of upper-limb gains by follow-up, on the other hand, it has been suggested that task-specific repeated practice regimens induce lasting motor cortical reorganization that often precedes motor improvement [42]. We may not yet be sure of the complex interplay between exercise frequency, intensity, and duration needed to optimize recovery, but the results would suggest that extending treatment duration could allow sufficient time for motor cortical reorganization to make way for motor recovery of the upper limb.

### Virtual Rehabilitation as an Additional Tool in the Management of Chronic Poststroke Upper-Extremity Recovery

Home-based intervention programs have been used in prior studies [43] as a central component of chronic poststroke study design. In this study, we included a control group that received an evidence-based standardized exercise program targeting the repetition of upper-limb movements that emulate ADL, which is currently frequently provided in rehabilitation programs. As such, participants in both groups benefited from a program that allowed for comparable levels of upper-limb activity.

While treatment group participants required a home installation of the Jintronix system, the setup was relatively simple, requiring minimal space in the participant’s living spaces and little to no technical maintenance throughout the duration of the intervention. A certain degree of computer literacy was required of the participants to navigate the interface, which was addressed during the first meeting. Furthermore, the system had the added capacity to inform clinicians of participant progress and time spent on the activities, factors which appeared to play an important role in recovery.

The treatment group participants played for a median of 527 minutes of activity (approximately 9 hours). Most participants needed no extrinsic prodding to engage in the program, perhaps relying instead on their desire to engage in the visually rewarding gaming environment [32]. We would argue that the intuitiveness of VR game consoles facilitates self-directed behavior, which ultimately influences program engagement and adherence, in line with the positive connection to the gaming avatar participants reported in a Nintendo Wii environment [44]. On the basis of these considerations along with the technology’s simplicity of use and installation, these findings support the feasibility of using VR serious games as tools for the management of chronic poststroke recovery, as recommended in the Guidelines for Adult Stroke Rehabilitation and Recovery [45].

### Study Limitations and Future Directions

Although the outcome measures included had strong psychometric properties, there were some limitations. The FMA-UE measure may have limited ability to detect changes when participants are either low or high on baseline arm function. The SIS and MAL measures may lack sensitivity in detecting smaller changes in self-perceived function. In addition, the use of subgrouping strategies to compare differences in baseline FMA-UE scores limited the ability to detect significant effects given the small overall sample size.

Participants in the standard care group did not log specific time spent on the GRASP program. More precision could have been achieved had wearable sensor technology been available and integrated into the program, which could have accurately kept track of time spent on active movements of the upper limb.

Participants in both groups gained significant arm function improvements while they were actively engaged in 1 of the 2 programs. Rather than draw comparisons between groups, future studies could set out to evaluate novel programs against a standardized one from a perspective of noninferiority, so that clinicians have more tools available to choose from for UE rehabilitation.

Future studies could further explore the impact of extended treatment time and increased number of visits for a follow-up evaluation to more easily keep track of changes in arm function. This would allow the possibility to test the increasingly popular theory that an increase in dosage results in better functional outcomes.

Finally, artificial intelligence could eventually be incorporated into VR serious games to reduce clinician involvement by adjusting difficulty level, movement range, and type of activities based on the user’s needs and preferences.

### Conclusions

There were visible trends of improvement following intervention for both interventions, particularly when participants were most actively engaged with the system. Depending on the individual and clinical context, the results indicate that VR serious games with clinician monitoring may be additional, effective, and feasible tools to include in the long-term management of upper-limb rehabilitation after stroke.

## Appendix

Multimedia Appendix 1 CONSORT-eHEALTH checklist (V 1.6.1).

## Abbreviations

ADL: activities of daily living
FMA-UE: Fugl-Meyer Assessment for upper extremity
GRASP: Graded Repetitive Arm Supplementary Program
MAL: Motor Activity Log
MCID: minimal clinically important difference
REDCap: Research Electronic Data Capture
SIS: Stroke Impact Scale
UE: upper extremity
VR: virtual reality
